# The role of leadership in enhancing non-technical skills in healthcare: a qualitative study in a Balkan context

**DOI:** 10.1186/s12960-025-01022-2

**Published:** 2025-10-13

**Authors:** Ruhija Hodza-Beganovic, Peter Berggren, Samuel Edelbring

**Affiliations:** 1 School of Health Sciences, Faculty of Medicine and Health, Örebro University, Örebro, Sweden; 2 International Medical Program, Center for Disaster Medicine and Traumatology, Department of Biomedical and Clinical Sciences, Linköping University, Linköping, Sweden; 3 School of Education, Culture and Communication, Mälardalen University, Västerås, Sweden; 4 Department of Computer and Information Science, Linköping University, Linköping, Sweden

**Keywords:** Leadership, Teamwork, Non-technical skills, Healthcare, Low- and middle-income countries, Balkans

## Abstract

**Background:**

Leadership is widely recognized as essential for fostering collaborative healthcare teams and improving patient outcomes. However, there is limited research on how leadership supports the development of nonclinical skills in healthcare settings in many low- and middle-income countries, including those in the Balkan region. This study addresses that gap by examining how leadership roles and practices enhance non-technical skills (NTSs)—such as communication, teamwork, and role clarity—among healthcare workers in the Balkans while also considering sustainable development, organizational values, cultural influences, and social dynamics.

**Methods:**

A qualitative approach was employed, drawing on data collected from three workshops conducted between 2018 and 2022 in university hospital clinics in Bosnia and Herzegovina, Kosovo, and Montenegro. Data sources included observations of group discussions, focus groups, and semi-structured interviews with healthcare leaders. Reflexive thematic analysis was used to identify patterns and develop key themes.

**Results:**

Four key themes emerged regarding the role of leadership in the development of NTSs: (1) defining roles and responsibilities, (2) fostering communication and teamwork, (3) promoting readiness for change, and (4) developing leadership competencies.

The participants noted that clear role definitions enhanced team coordination, inclusive communication reduced misunderstandings, supportive leadership eased resistance to change, and mentorship served as a valuable mechanism for leadership development.

**Conclusion:**

Leadership plays a key role in strengthening NTSs in Balkan healthcare contexts by promoting communication and teamwork within culturally and hierarchically complex environments. Role clarity, open dialogue, and shared accountability emerged as key factors for effective team performance and patient safety. These findings highlight the need for leadership development and the implementation of formal training initiatives—such as structured mentorship programs—to foster collaborative and resilient healthcare systems in low- and middle-income countries.

**Supplementary Information:**

The online version contains supplementary material available at 10.1186/s12960-025-01022-2.

## Background

Effective leadership is fundamental to building functional healthcare teams, maintaining organizational stability, and ensuring patient safety. While leadership is widely recognized in high-income countries as a key driver of collaborative work environments and improved clinical outcomes, there is comparatively less research on its role in low- and middle-income countries (LMICs)—particularly in supporting the development of non-technical skills (NTSs) within healthcare settings [1–4].

NTSs, encompassing cognitive, social, and personal competencies, support effective clinical work by complementing clinical expertise and enhancing service delivery and interprofessional coordination. These skills include situational awareness, decision-making, communication, teamwork, leadership, and stress management [5]. Among them, leadership plays a pivotal role in shaping team dynamics and fostering effective collaboration [4, 5]. In LMICs, where formal management structures are often underdeveloped and healthcare systems are challenged by workforce shortages, limited resources, and rigid hierarchies [3, 4, 6], strong leadership is especially critical. Effective healthcare leadership in these contexts is characterized by clearly defined roles, collaborative teamwork, inclusive communication, and a commitment to team development [6].

In many parts of the Balkans, healthcare systems have traditionally been centralized and hierarchical, with senior physicians dominating decision-making processes [1, 2]. Although participatory leadership—characterized by shared decision-making and team engagement—is gradually emerging, its adoption remains limited. This is largely due to persistent hierarchical norms, weak interprofessional collaboration, and the lack of structured leadership training [1, 6, 7]. However, evidence from both global and LMIC contexts indicates that participatory leadership can significantly enhance team cohesion, trust, and quality of care, particularly when supported by institutional readiness and aligned policy frameworks [6–8].

Despite growing recognition of leadership’s importance in improving healthcare outcomes, LMICs continue to face substantial gaps in leadership education and capacity-building [1, 4, 6]. Many healthcare leaders lack formal training, resulting in inconsistent decision-making and the persistence of hierarchical cultures [3, 6, 7]. Although targeted efforts—such as leadership training programs in Bosnia and Herzegovina—have sought to enhance team coordination through structured development frameworks [2], such initiatives remain limited. These examples highlight the value of context-specific strategies that support the application of NTSs in complex care environments and underscore the need for continued research in this area [9, 10].

Leadership theory offers valuable insights into how leadership shapes team dynamics and motivation. Kozlowski et al. [11] highlighted its influence on team performance and emphasized its role in enabling experiential learning within teams [11–13]. Bass et al. [14] described transformational leadership as a style that motivates individuals to exceed expectations, while participatory leadership emphasizes shared decision-making and team involvement. Burke et al. [13] distinguished between task-focused leadership, which supports operational efficiency, and person-focused leadership, which fosters team cohesion. In this study, leadership is understood both as a set of behaviors enacted by healthcare leaders [13, 15] and as a developmental process in which competencies are built over time through continuous learning [11, 13].

Given this context, this study explores how leadership contributes to the development of NTSs among healthcare professionals in the Balkan region. It examines the experiences and perceptions of healthcare leaders and multidisciplinary team members, with particular attention to the organizational and cultural conditions that shape leadership effectiveness. This qualitative approach provides an in-depth understanding of leadership dynamics and illustrates how the local context influences collaboration and professional growth.

The following research questions guide this study: (1) What defines leadership in an LMIC healthcare context in terms of key characteristics, roles, skills, and responsibilities? (2) How does leadership support the professional growth and sustainable development of healthcare team members? (3) How is leadership perceived from an inside-out perspective (the leader’s view) versus an outside-in perspective (the team members’ views)? To address these questions, we explored leadership practice and development over time, drawing on perspectives from participants and leaders collected across three sequential workshops in the region.

## Study design and methodology

**Fig. 1:**
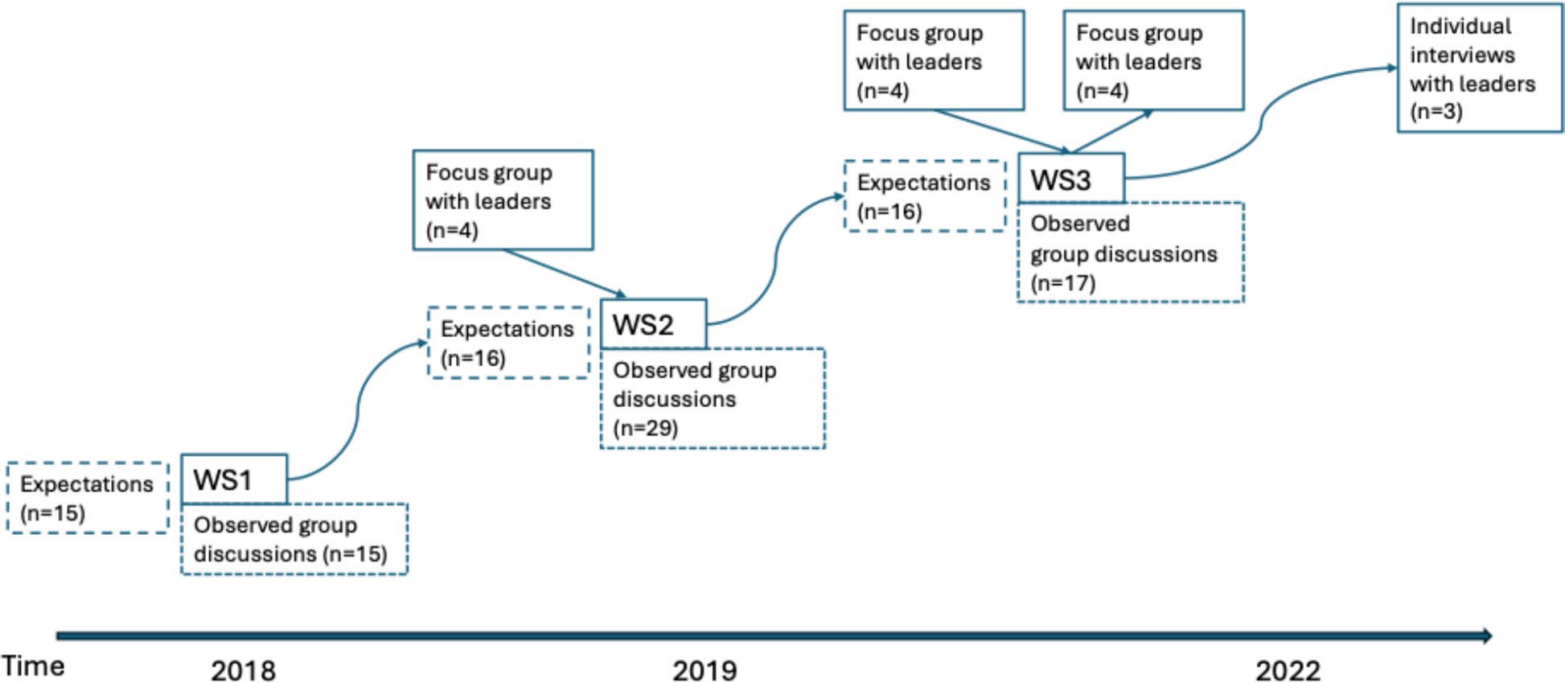
Overview of the data collection and analysis

This study adopted a qualitative research design using multiple data collection methods. Participants were recruited from three university hospital clinics located in Sarajevo (Bosnia and Herzegovina), Pristina (Kosovo), and Podgorica (Montenegro). They were invited to participate in a series of three interactive workshops, held at shared locations in the Balkans between 2018 and 2022, aimed at strengthening NTSs among multidisciplinary healthcare teams [16] (Fig. 1). Although the workshops were initially planned within a shorter timeframe, the Covid-19 pandemic caused delays in scheduling and coordination across sites. The workshops were collaboratively designed by local healthcare leaders, researchers, and international healthcare developers based in Sweden [17].

A total of 29 participants attended Workshop 2, with the same group—although slightly smaller—participating in Workshops 1 and 3. This continuity enabled the collection of longitudinal qualitative data on leadership practices in clinical settings, along with participant reflections on leadership development. The design allowed us to examine how understandings and enactment of leadership evolved over time within the unique sociocultural and organizational context of the Balkans.

Participants included both physicians and nurses (see Table 1), with formal leadership roles held by physicians.

**Table 1:** Background of the workshop participants

	WS1	WS2	WS3
Number of participants	15	29	17
Observed group discussions	Three group discussions with five persons each	Three group discussions with nine-ten persons each	Three group discussions with five or six persons each
Age (years), mean (SD)	47.8 (8.5)	47.4 (8.9)	51.4 (9.4)
Gender	Male, *n* = 7	Male, *n* = 11	Male, *n* = 8
	Female, *n* = 8	Female, *n* = 18	Female, *n* = 9
Professional background	Physicians, *n* = 8*	Physicians, *n* = 14*	Physicians, *n* = 9*
	Nurses, *n* = 7	Nurses, *n* = 15	Nurses, *n* = 8

^*^Healthcare leaders are included within the physician group

Three clinic heads participated in all three focus group discussions and follow-up interviews. A fourth senior healthcare leader took part in the first two workshops but retired before Workshop 3 and was, therefore, not included in the final round. Although this change slightly reduced the number of perspectives, it did not compromise continuity within the leadership group.

### Data collection

The workshops provided a structured setting where the participants engaged in facilitated learning, reflection, and peer dialogue. Although we did not directly observe leadership behaviors in clinical practice, the research team gained insights into the clinical context through narratives shared by leaders and participants. This indirect approach allowed the study to explore both how leadership was understood and how it was enacted from the participants’ own perspectives.

#### Observed group discussions

Group discussions captured the workshop participants’ experiences and perspectives on leadership and introduced NTSs. Field notes were recorded by members of the research team (see Table 1) [18].

#### Focus groups and interviews with leaders

Three clinic heads, along with the healthcare leader who retired between Workshops 2 and 3, participated in the workshops and three interactive focus group discussions held at key time points: before the second and third workshops and after the final workshop. These focus groups captured leaders’ perceptions of NTSs and the role of leadership in ensuring the sustainability of NTSs. Field notes were recorded by members of the research team and two independent observers.

Individual semi-structured interviews were conducted with three leaders to obtain their in-depth personal perspectives [19]. With permission, the interviews were audio-recorded and transcribed verbatim to ensure the accuracy of the data. Two interviews were conducted face-to-face at the leaders’ workplaces, while one was conducted via Zoom.

The focus groups and interviews with healthcare leaders aimed to explore key areas related to leadership practice and gain insights into the leaders’ understanding of NTSs, how leadership was enacted in practice and how it evolved over time. A semi-structured interview guide covering these areas, along with example questions was used across all focus groups and interviews (Supplementary material 1).

#### Participants’ written expectations and researchers’ observations

Prior to each workshop, the participants were asked to compose a brief email outlining their expectations for the upcoming session, providing qualitative insights into what they hoped to gain. In addition, during each workshop, members of the research team and two independent observers took field notes on group dynamics, participant engagement, and the content of group presentations and activities.

### Analysis

All qualitative data sources were organized and securely stored. The transcripts comprised over 50 pages of text, supplemented by detailed field notes. The data were analyzed using a reflexive thematic analysis approach [20], allowing themes to emerge inductively without imposing predefined categories. Attention was given to the participants’ professional roles to explore variations in perspectives based on background. The research team adopted a constructivist orientation, recognizing that meaning is co-constructed by participants and researchers rather than objectively discovered [21].

The analysis unfolded through several iterative stages. The first author transcribed the interviews and reviewed them alongside the field notes and the participants’ written expectations. Initial codes were openly developed and refined through repeated cycles of analysis and team discussions. Themes were iteratively reviewed and adjusted until a coherent and meaningful structure emerged [20].

Individual researcher perspectives served as valuable analytic resources throughout the thematic analysis. The first author (RHB) contributed contextual insight from her clinical experience in the Balkans, supporting interpretation of participant reflections. PB brought experience from international development projects in the Balkans, while SE offered a pedagogical perspective from health professions education. These complementary viewpoints fostered reflexive dialogue, enhancing the interpretation of participant narratives and strengthening analytical rigor.

In the final analytic phase, themes were clearly defined and situated within the broader socio-organizational and cultural context of Balkan healthcare. This included reflection on how hierarchical team structures, limited resources, and entrenched professional norms shaped leadership practices. The first author’s contextual knowledge, supported by regular discussions among researchers with both insider and outsider perspectives, ensured that the analysis was both grounded and theoretically robust.

When defining and naming themes, we discussed how each theme reflected recognized features of healthcare in the Balkans, such as hierarchical structures and resource constrains. To stregthen contextual validity, we also considered the researchers’ experiential knowledge of the Balkan region and relevant insights from the literature.

## Results

The analysis resulted in four themes that captured the key leadership dimensions related to NTS development:

1. Defining roles and responsibilities – leadership clarifies healthcare workers’ responsibilities.
2. Communication and teamwork – leadership fosters open communication and interprofessional collaboration.
3. Readiness for change – leadership balances traditional hierarchies with participatory practices to support change.
4. Leadership competency development – leadership skills are acquired through experiential learning and mentorship.

Throughout the workshops, healthcare workers and leaders reflected on the value of structured leadership in creating stable teams, clarifying roles, fostering inclusivity, and enabling shared decision-making. However, the development and enactment of NTSs, including communication, collaboration, and adaptability, were shaped by deeply embedded contextual factors, such as organizational hierarchies and cultural norms specific to the Balkan healthcare setting.

Theme 1: Defining roles and responsibilities – leadership clarifies healthcare workers’ responsibilities.

Across all three clinics, the participants emphasized role clarity in aligning individual and team goals. Early in the project, they described significant ambiguity around responsibilities, which led to inefficiencies and overlapping tasks. As one nurse noted during the first workshop,

“I expect that leaders will be supportive so that each member has a clear position, role, and tasks while also creating a positive working atmosphere and working habits.” (Nurse, WS1)

Similarly, a physician noted:

“Sometimes we assume that everyone knows their role, but when things go wrong, it becomes clear that this is not the case. We work as individuals rather than as a team, and that is when errors happen.” (Physician, WS1)

These reflections highlighted the critical role of leadership in defining and communicating responsibilities. As the workshops progressed, the participants increasingly recognized that effective leadership was essential for establishing structured routines and fostering shared accountability.

One team member later explained,

“When we know our role and responsibilities, we function as a system rather than as isolated individuals. It prevents duplication of tasks and ensures that critical duties do not get overlooked.” (Focus group, WS2)

This shift reflected a growing commitment to leadership practices that promote clarity, coordination, and enhanced team performance.

Theme 2: Communication and teamwork – leadership fosters open communication and interprofessional collaboration.

Participants in all clinics reported persistent communication challenges, particularly between professional groups and across clinical boundaries.

As one physician explained,

“Communication about joint patient treatment between clinics is sometimes one-way, so that treatment plan and expected outcomes are not always shared.” (Physician, WS1)

Differences in clinical judgment further illustrated gaps in shared understanding:

“One physician thinks the risk is high, while the other that it is moderate. In that case, the lab results or other findings would be used to reevaluate the case and reconsider the risk.” (Nurse, WS1 discussion)

The nurses also described being excluded from decision-making:

“We are expected to carry out the treatment plan, but we are not always informed about the reasoning behind decisions. Sometimes, by the time we get information, it’s too late to make changes.” (Nurse, WS2)

Over time, the clinics introduced clearer communication routines, including regular team briefings and increased nurse involvement in meetings. These adjustments helped address earlier gaps and promoted more coordinated care.

As one leader reflected in a later phase,

“Previously, staff members would approach me directly, but now these discussions occur in designated meetings with the main nurse. We believe it is crucial for difficulties and concerns to be addressed so that everyone is informed about the situation.” (Leader 2, WS3)

These changes reflected a growing understanding among leaders that open, consistent communication was essential for safe and coordinated care.

Theme 3: Readiness for change – leadership balances traditional hierarchies with participatory practices to support change.

Many participants expressed openness to change, but described organizational resistance, often rooted in entrenched hierarchies.

As one physician explained,

Change is difficult because the system itself resists it. When you try to introduce new ways of working, you often hear, “This is how we’ve always done it.” (Physician, WS2)

The leaders acknowledged this challenge:

“We must balance tradition with innovation. If we push too hard, we create resistance; if we do nothing, we stagnate.” (Leader 1, WS3)

“Transitions are often more readily accepted when advocated by outside stakeholders and when they were introduced in a way that respected existing workplace structures.” (Leader 2, WS3)

Over time, the participants pointed to positive examples of change. The urodynamic team was frequently cited as a model of interprofessional collaboration.

“The team helped narrow the gap between professions. It showed that it is possible to work together effectively.” (Focus group, WS2)

The leaders described how their team introduced structured interdisciplinary meetings, shared documentation, and active nurse participation in planning. This not only shifted the leadership culture toward greater participation but also improved patient engagement and team trust.

Ultimately, leadership that was both supportive and adaptive proved essential in enabling change while navigating organizational tradition.

Theme 4: Leadership competency development – leadership skills are acquired through experiential learning and mentorship.

A consistent theme was the absence of formal leadership training. Most participants reported learning leadership through experience or mentorship, rather than structured programs.

“We are expected to lead without ever being taught how to lead. You learn as you go, and sometimes that means making mistakes.” (Leader 3, WS1)

“The best leadership training I had was learning from someone I respected. We need more structured mentorship programs that allow younger professionals to learn leadership skills in a supportive way.” (Leader 2, WS2)

The physician stressed the need for leadership that empowers others.

“Ensure that team members and leadership competencies are improved. Leaders should not be the ones who strictly have to make decisions—they have to facilitate and support the team in making decisions.” (Physician, WS1)

Through ongoing reflection during the workshops, the leaders began adopting more participatory behaviors, recognizing the value of listening and shared problem-solving.

“I changed my mindset and returned home with new ideas. We are inspired on how to work together. I am open to listening, thinking, and accepting others’ opinions not only because it is my responsibility but because the staff are tired and frustrated.” (Leader 1, interview).

These reflections underscored how leadership competencies are not static but evolve through exposure, reflection, and interaction in real-world clinical settings.

## Discussion

This study explored the role of leadership in fostering NTSs among healthcare professionals within the Balkan context, focusing on how leadership is defined, enacted, and perceived by team members and formal leaders. Our findings provide insights into leadership practices in LMICs and illustrate how leadership functions both as a stabilizing force and a catalyst for change. The results demonstrate that effective leadership is not solely dependent on formal authority or administrative structures but is a dynamic, context-sensitive process shaped by organizational culture, team dynamics, and systemic constraints. Leadership plays a pivotal role in bridging the NTS gap by creating an environment where healthcare professionals can develop and apply these skills in daily practice.

These findings must be interpreted within the broader socio-organizational context of Balkan healthcare systems, which remain shaped by hierarchical structures and legacy administrative cultures. While participatory leadership is gaining recognition, its adoption is challenged by rigid institutional frameworks, limited leadership development programs, and persistent role boundaries between professions. A key aspect of this transition involved shifting perceptions of patient safety and risk management from individual responsibilities to shared team functions. As this shift toward collective accountability occurred, it helped reduce the prevailing blame culture. These findings underscore the essential role of leadership in enabling this change and align with Scott et al.’s [4].

Regarding research question 1, leadership was understood as both a formally assigned and an informally assumed function within healthcare teams. In the Balkan context, while hierarchical norms persisted, a gradual shift toward inclusive and transformational leadership [14] was evident. Leaders who acted as local change agents effectively introduced new ideas and strengthened collaboration [12, 22]. These findings align with transformational leadership theory, which emphasizes vision, empowerment, and intellectual stimulation [14]. During the workshops, collaborative leadership styles enhanced engagement and adaptability, reflecting Salas et al.’s emphasis on shared responsibility [23].

Despite this potential, several barriers hindered leadership development, including limited institutional support, misalignment with local norms, and a lack of formal training opportunities. The persistence of hierarchical structures often restricted mentorship and open communication. These findings support previous calls for context-sensitive leadership development tailored to LMIC healthcare systems [3, 24].

Importantly, the study highlighted the need for organizational readiness to embrace change. Leaders with backing from upper management and external actors (e.g., policymakers and NGOs) were more successful at initiating and sustaining leadership initiatives. This supports Gordon et al.’s [25] assertion that successful leadership transitions require alignment between internal and external stakeholders.

In addressing research question 2, we found that the leaders supported communication, facilitated knowledge sharing, and encouraged continuous development. These roles were instrumental in clarifying responsibilities and reducing interprofessional conflict. Our findings align with inclusive leadership models and Flin et al.’s [5, 9] and Reeves et al.’s [24] studies, which highlighted the role of leadership in improving team performance and safety.

Structured workshops served as effective forums for practicing leadership behaviors. In settings with deeply ingrained hierarchies, these workshops provided a space for inclusive dialogue and skill-building. Active nurse participation further demonstrated the potential to broaden leadership across professions. These findings echo the principles of team leadership described by Salas et al. [23] and Burke et al. [13].

In addressing research question 3, the perceptions of leadership varied significantly between leaders (“inside-out”) and team members (“outside-in”). While both groups acknowledged the value of leadership for team effectiveness and professional development, the leaders focused on formal responsibilities and top-down communication, consistent with transactional leadership, prioritizing structure, oversight, and role delineation [11, 13]. In contrast, the team members emphasized shared leadership, supportive behaviors, and inclusivity, reflecting preferences for participatory and team-based approaches [15].

Overall, this study shows that effective leadership in LMICs is both relational and developmental. It fosters role clarity, communication, and continuous learning—critical elements for the development of NTSs. While the formal leaders highlighted structural limitations, the participants emphasized leadership that promoted shared accountability and psychological safety. These findings support the need for ongoing reflection, mentorship, and leadership development processes tailored to the sociocultural realities of LMICs [22, 23, 25].

However, this study has several limitations. First, although both nurses and physicians participated in the workshops, formal leadearship roles and consequently the interviews were dominated by physicians. This limited the inclusion of perspectives from other healthcare professionals, such as nurses in leadearship positions. Second, the small sample size and limited geographic scope constrained the generalizability of the findings. Although the qualitative design offered in-depth insights, we did not evaluate clinical or organizational outcomes, which limits conclusions about practical impact. Third, the Covid-19 pandemic delayed the workshop schedule, affecting planning and continuity of participant involvement. Although most participants attended multiple workshops, a few attended only once, which may have reduced our ability to trace changes in understanding over time.

## Conclusions

This study demonstrates that leadership plays a important role in developing NTSs and strengthening teamwork in resource-constrained healthcare settings. By clarifying roles, promoting communication, and fostering shared accountability, leadership can enhance collaboration and may improve patient safety. The findings highlight the importance of inclusive, team-based leadership practices in LMICs and the need for formal leadership development initiatives—including mentorship and reflective practice—tailored to the cultural and organizational realities of these settings. Leadership development should focus not only on individual competencies but also on creating the enabling environment necessary for effective, collaborative healthcare delivery.

## Supplementary Information

Supplementary material 1.

## Data Availability

Further data can be requested from the corresponding author through reasonable requests.

## Abbreviations

NTSs: Non-technical skills
LMICs: Low- and middle-income countries
