# Supplementary material for: The role of leadership in enhancing non-technical skills in healthcare: a qualitative study in a Balkan context
Source: Hum Resour Health. 2025 Oct 13;23:53. doi: 10.1186/s12960-025-01022-2 (PMC12516859; doi:10.1186/s12960-025-01022-2)
Supplement: Supplementary file 1 — Supplementary material 1. [file 12960_2025_1022_MOESM1_ESM.docx]

## Supplementary Material 1

### Semi-structured interview guide for focus groups and interviews with leaders

This guide outlines a semi-structured interview protocol used for data collection in focus groups and individual interviews with healthcare leaders. Example questions are grouped into categories aligned with the study's research objectives.

### 1. Leadership practice and support for Non-Technical Skills (NTS)

- *Have you made any changes in how you manage your clinic as a result of the workshops?*
- *Are there any formal or informal opportunities for training related to NTS?*
- *Do you think NTS are relevant for clinical work?*
- *Do you find that the knowledge from the workshops is applicable in your organization?*
- *Have you observed any improvements in your clinic related to NTS since the workshops?*
- *Do others in your organization seek to learn from your experience with the workshops?*

### 2. Supporting professional development and defining roles and responsibilities

- *How is professional development organized in your clinic or hospital?*
- *How are roles and responsibilities defined and communicated in your team?*
- *How is responsibility shared within the team?*
- *What typically happens when an error occurs in the clinical setting?*
- *Who is responsible for ensuring tasks are completed correctly?*
- *Does organization support a continuous learning and development intentions?*

### 3. Leadership (support, training) and organization challenges

- *Do you find it challenging to have a leadership role?*
- *Do you find it challenging to involve your staff in making decisions?*
- *Are there any organization challenges that influence your ability to implement or enhance NTS?*
- *Does your clinic or hospital management support teamwork and interprofessional collaboration?*

### 4. Communication within and across teams

- *What are the usual ways of communication between team members and leadership? (e.g., meetings, reports)*
- *How are patients discussed and decisions made? What if there are differences in opinion?*
- *Is open communication with leadership encouraged or practiced?*

### 5. Teamwork in daily practice

- *Is teamwork a regular part of your daily work? Do you have specific routines or structures when working in teams?*
- *Do the teams consist of professionals from different backgrounds?*
- *Do you work with the same team consistently or with different teams?*
- *Have you observed any specific changes in your team since participating in the workshops?*
